# The Evolutionary History and Morphological Divergence of Corallivorous Fishes on Coral Reefs

**DOI:** 10.1093/iob/obag032

**Published:** 2026-08-08

**Authors:** I Marrable, N Peoples, M A Kolmann, J M Huie, W F Figueira

**Affiliations:** School of Life and Environmental Sciences, The University of Sydney, Camperdown, Sydney, NSW 2050, Australia; Department of Evolution and Ecology, University of California, Davis , Davis, CA 95616, USA; Department of Biology, University of Louisville, Louisville, KY 40292, USA; Department of Ecology and Evolutionary Biology, University of California, Irvine, Irvine, CA 92697, USA; School of Life and Environmental Sciences, The University of Sydney, Camperdown, Sydney, NSW 2050, Australia

## Abstract

Specialized ecological niches can represent evolutionary traps that increase extinction risk when environments change. The consumption of coral tissue, mucus, and skeleton is a rare feeding niche despite the abundance of coral on reefs, which raises questions about the evolutionary consequences of this dietary specialization. In this study, we used phylogenetic comparative methods, micro-CT scanning, and scanning electron microscopy to explore the evolutionary history of corallivory across reef fishes and compare feeding morphology across species within a major clade of corallivorous species (Chaetodontidae) that vary in their coral consumption. We show that obligate corallivory has evolved independently at least 17 times but is an evolutionary “dead end” for most reef fish species. Early origination of facultative corallivory may have contributed to the success of butterflyfishes, the predominant clade of coral-feeding fishes, as they diversified in competitive reef environments. Obligate and facultative butterflyfish species differ primarily in dental morphology while overall cranial traits are largely similar between groups. Coral feeding requires continuous foraging with narrow energetic margins, and climate-driven disturbances occur markedly faster than evolutionary transitions away from corallivory. This temporal mismatch between change at ecological and evolutionary timescales subjects corallivorous fishes to heightened vulnerability as climate-driven disturbances increase in frequency and severity.

## Introduction

Morphological change to the feeding system often accompanies transitions to new diet niches. In some environments, heightened competition between species can catalyze the evolution of this morphological novelty and spur movement from generalist to specialized niches (Martin and Wainwright 2013; Carscadden et al. 2023). However, the evolutionary dynamics of ecomorphological specialization can be complex (Futuyma and Moreno 1988). Specialized feeding niches often require a set of unique morphological adaptations in the feeding apparatus to maintain position on a narrow fitness peak, even though the resource itself may be abundant. Often, these traits evolve rapidly within species occupying a specialized niche (Martin and Wainwright 2011; Borstein et al. 2018). Specialization can lead to evolutionary irreversibility of ecological niches, acting as an evolutionary “dead end” if the specialized adaptations cannot be co-opted for alternative ecologies (Futuyma and Moreno 1988; Moran 1988; Raia et al. 2016). Support for this dead-end hypothesis in macroevolutionary studies is mixed (Vamosi et al. 2014; Day et al. 2016). On one hand, ecological specialization (e.g., herbivory) can provide a basis from which future diversity can arise by increasing diversification rates (Price et al. 2012) whereas generalization (e.g., omnivory) can act as a macroevolutionary sink (Burin et al. 2016). However, ecological specialization can also significantly reduce or restrict entirely subsequent transitions into alternate diet niches (Lobato et al. 2014; Egan et al. 2018). These lineages may therefore be more vulnerable to extinction if they cannot keep up with a changing environment or the accessibility of their preferred resources changes (Van Valen 1973; Devictor et al. 2010; Dennis et al. 2011; Wright et al. 2013; Spiridonov and Lovejoy 2022). For example, hypercarnivory in large mammals requires a suite of irreversible dental modifications that limit transitions to more generalized diets, leading to ecological irreversibility and positioning clades on a trajectory of eventual decline (Van Valkenburgh 1989, 1991; Holliday and Steppan 2004; Balisi and Van Valkenburgh 2020). This raises questions about the prevalence of irreversible ecological niches, particularly those in environments susceptible to rapid change, and the morphological adaptations needed to access them.

Tropical coral reef ecosystems are emblematic centers of biodiversity and support a diverse assemblage of ray-finned fishes (Actinopterygii) (Alfaro et al. 2007; Cowman and Bellwood 2011). The evolution of fishes that inhabit coral reefs represents multiple independent invasions into resource rich but increasingly saturated habitats (Price et al. 2014). Accordingly, reef fishes have evolved a wide range of feeding adaptations in order to fill an array of ecological niches. These include feeding on coral mucus and polyps (Motta 1989; Huertas and Bellwood 2018), ectoparasites (Baliga and Mehta 2015, 2019; Huie et al. 2020), turf algae, and particulate material within (Bellwood et al. 2014; Tebbett et al. 2018; Mihalitsis and Wainwright 2024), hard-shelled prey (Turingan and Wainwright 1993), zooplankton (Floeter et al. 2018; Ng et al. 2025), and fish (Mihalitsis and Bellwood 2019). However, the number of lineages occupying these niches does not reflect the abundance of the resource itself. For example, up to 53% of coral reef fishes may be piscivorous owing to the abundance of fish prey (Randall 1967; Hixon 1993) yet just 128 species (∼2% of reef fishes) incorporate some amount of coral in their diet despite coral making up the structure of reef habitats (Cole et al. 2008). Shifts to feeding on low-quality, but abundant, food resources such as coral may allow lineages to diversify in competitive reef environments (Lobato et al. 2014), but the evolutionary ease at which corallivory evolves and is lost across reef fishes, and how this may predispose species to a heightened extinction risk on modern reefs, is not known.

Corallivory is the consumption of coral tissue and/or skeleton to meet all or a portion of an organism’s nutritional demands, and corallivorous fishes are present in all tropical coral-dominated ecosystems (Cole et al. 2008; Rotjan and Lewis 2008; Bellwood et al. 2010; Huertas and Bellwood 2018). In addition to fishes, corallivores on coral reefs include many invertebrates such as starfish (e.g., crown-of-thorns starfish, *Acanthaster planci*) snails (e.g., *Corallophila* spp.), crabs (e.g., *Tetralia* spp., *Trapezia* spp.), and urchins (e.g., the slate pencil urchin, *Eucidaris thouarsii*) (Glynn 1973; Stimson 1990; Rotjan and Lewis 2008). Within fishes, corallivores can be delineated by the mode of coral feeding. Small-bodied pickers (e.g., butterflyfishes) remove superficial mucus, polyps and tissue while larger-bodied species (e.g., parrotfishes, *Arothron*) remove tissue and calcareous skeleton together, either through targeted, deep “excavating” bites (e.g., *Chlororus*) or dispersed “scraping” bites (e.g., *Sparisoma*) (Bellwood and Choat 1990; Floeter et al. 2018). However, recent evidence indicates that some larger corallivores, particularly parrotfishes, are more likely “indirect” corallivores that target cyanobacteria and microorganisms on calcareous substrate rather than coral directly (Clements et al. 2016; Nalley et al. 2022), whereas smaller-bodied corallivores (e.g., *Chaetodon*) target and assimilate coral resources (Harmelin-Vivien and Bouchon-Navaro 1983; Pratchett 2007). Coral is an inherently difficult resource to utilize because stinging nematocysts cover a sharp calcium carbonate skeleton, and the accessible tissue layer is often thin. To overcome this, corallivorous fishes may have evolved adaptations to the feeding system. The Tubelip wrasse (*Labropsis australis*) has foliose mucus producing lips that may provide a physical barrier against stinging nematocysts, thereby allowing individuals to feed directly upon coral tissue (Huertas and Bellwood 2017); this novelty is not seen in related non-corallivorous species (Huertas and Bellwood 2018). Other corallivores such as the guineafowl puffer (*Arothron meleagris*) use a beak-like dentition to scrape into the carbonate skeleton or bite tips of branching coral directly (Hiatt and Strasburg 1960; Glynn et al. 1972). The intramandibular joint present in multiple reef fish clades, including those with corallivorous species, may provide a functional advantage during biting on uneven surfaces such as calcium carbonate skeletons (Konow and Bellwood 2005; Gibb et al. 2015; Konow et al. 2017).

Butterflyfish, bannerfish, and coralfish (Chaetodontidae) are abundant on coral reefs and account for 52% of all corallivorous fishes (Cole et al. 2008; Rotjan and Lewis 2008; Bellwood et al. 2010). Butterflyfishes vary substantially in the nature of corallivory. Some species are obligate coral feeders, targeting specific coral species while others opportunistically incorporate a range of coral resources into their diet or do not consume coral (Pratchett et al. 2004; Pratchett 2005, 2007; Cole et al. 2008; Rotjan and Lewis 2008; Coker et al. 2023). However, investigation into the morphological differences between butterflyfishes that vary in their affinity for coral are limited (Motta 1988, 1989). In this study, we first use phylogenetic comparative methods to evaluate the evolutionary history of corallivory across reef fishes. Our objectives were to (1) assess if transitioning into a corallivorous niche represents an evolutionary dead end in the history of reef fishes, and (2) to investigate whether obligate, facultative, and non-corallivorous butterflyfishes differed in feeding morphology. Using and ecological classification of coral-feeding fishes, we estimate evolutionary transition rates between modes of corallivory both across all reef fishes and separately within Chaetodontidae. We then use micro-CT scanning and scanning electron microscopy (SEM) to evaluate differences in feeding morphology between 17 butterflyfish species that vary in their reliance on coral as a food resource. Finally, we frame the evolution of corallivory in the context of current climate change. Our work provides a new appraisal of the evolution of a rare trophic niche and the morphological adaptations needed to access it.

## Methods

### Diet ancestral state reconstruction

We performed an ancestral state reconstruction to quantitatively investigate the evolutionary history of corallivory across reef fishes. First, we summarized the distribution of 100 near-complete phylogenies from (Siqueira et al. 2020), which are based on the all-taxon-assembled chronogram of (Rabosky et al. 2018), using the “*mapTree*” function in *RevBayes* (Höhna et al. 2016) to generate a single maximum *a posteriori* (MAP) timetree. This timetree includes 6257 species of coral reef fishes. We used the list of corallivorous reef fishes from (Cole et al. 2008) to identify reef fishes as obligate (>80% of diet includes coral) and facultative (coral is supplemented with other invertebrates and algae) corallivores; all fishes absent from this list were coded as non-corallivorous. The list of Cole et al. (2008) was produced through a comprehensive literature survey that included gut contents, observations of coral feeding, and anecdotal evidence. As phylogenies built using stochastic polytomy resolvers should not be used for analyses of trait evolution (Rabosky 2015), we took an approach that maximizes inclusion of non-corallivorous species while ensuring evolutionary transitions were estimated from molecular subtrees. For every genus that included corallivorous species (e.g., *Chaetodon, Heniochus*, and *Labropsis*), we removed species that lacked molecular data from the phylogeny. If a corallivorous species lacked molecular data but was a singleton within a larger clade (e.g., *Exallias brevis*), we retained the species; the pruned tree included 6175 species. We fit Bayesian discrete character evolution models in RevBayes; we used a reversible-jump Markov Chain Monte Carlo (rj-MCMC) to directly detect irreversibility between diet states (e.g., transition rate away from state equal to 0) while simultaneously estimating transition rates for diet transitions that do occur. We included a “rates” model with rates drawn from an exponential distribution and a prior of 50 transitions across the phylogeny, and a “0” model in which all transition rates are fixed to 0. This framework effectively explores the entire model space of discrete character evolution models (e.g., ER, SYM, ARD, irreversible) by allowing each transition rate to be equal to any other rate, different from any other rate, or 0 with no a priori designation. We set a mixing prior of 0.5 (i.e., prior probability that a rate is equal to 0). We directly estimated the posterior probability (PP) that any transition rate is equal to zero (i.e., irreversible) by recording the frequency of the “0” model and considered a rate to be 0 when PP of 0 is greater than or equal to 0.75. We assumed the root to be in a non-corallivorous state, as corallivory is likely a recent feeding niche (Bellwood et al. 2010; Huertas and Bellwood 2018). We estimated transition rates and ancestral states using MCMC; the chain was run for 50,000 generations (tuning interval = 200) with 25% burn-in. Effective sample sizes (ESS) for all parameters were >9000, indicating that the MCMC chain effectively explored the posterior and autocorrelation between generations quickly breaks down. We used stochastic character mapping (Bollback 2006) to generate the MAP character history using the “*characterMapTree*” function in RevBayes. We processed the RevBayes outputs and plotted the ancestral state reconstruction using the R packages “*RevGadgets*” and “*phytools*” (Revell 2012; Tribble et al. 2022).

To ask whether transition rate patterns differed across reef fish clades, we repeated the analysis above within Chaetodontidae (butterflyfishes), the predominant clade of corallivores on coral reefs which contains multiple transitions between corallivorous states. By separately analyzing this subset of the reef phylogeny, we can identify whether the evolution of corallivory within Chaetodontidae may differ compared to general patterns across reef fishes. We pruned our full phylogeny to include only Chaetodontidae species with molecular data (*n* = 96) using the “*drop.tip*” function in the R package “*geiger*” (Pennell et al. 2014). We used the same reversible-jump MCMC framework described above; the unequal rates model had a prior of 30 transitions as fewer transitions likely occur within Chaetodontidae compared to across reef fishes, with a mixing prior of 0.5. We set the root state to facultative corallivory, as analyses across reef fishes identified the ancestor of Chaetodontidae to be a facultative corallivore (see Results). The MCMC was run for 50,000 generations (tuning interval = 200) with 25% burn-in; ESS >12,000 for all parameters.

### Comparative morphology of corallivorous fishes

We used micro-computed tomography (μCT) to assess how feeding morphology varies between obligate, facultative, and non-corallivorous species within Chaetodontidae. We examined six obligate corallivores representing three independent evolutionary origins of obligate coral feeding (Fig. 3) (*Chaetodon trifascialis, C. trifasciatus, C. aureofasciatus, C. ornatissimus, C. melannotus, and C. unimaculatus*), seven facultative corallivores (*Heniochus chrysostomus, Forcipiger flavissimus, C. auriga, C. ephippium, C. lunula, C. vagabundus, and C. capistratus*), and four non-corallivorous species (*Roa haraguchiae, Johnrandallia nigrirostris, C. humeralis*, and *Chelmon rostratus*) (Table S1). We acknowledge that, though including most genera, the low sample size (∼13% family-level sampling fraction) is a limitation of this study. We made two substitutions of taxa for species missing from the molecular phylogeny to accommodate phylogenetic analysis of variance (ANOVA) tests; *Roa haraguchiae* was represented by *Prognathodes aya*, and *Chaetodon humeralis* was represented by *Prognathodes falcifer*. These substitute taxa were chosen to preserve clade relationships. Whole specimens were scanned at a resolution of 18–21 μm voxel size (65 kV, 123 μA). Jaws were closed in a natural position so that dentition was interlocked. Scans were reconstructed in NRecon (Bruker Corp., Billerica, MA, USA), then scaled and rendered using the SlicerMorph plugin for 3D Slicer v5.6.2 (Fedorov et al. 2012; Rolfe et al. 2021); linear measurements were collected using the “line” tool in the Markups module. All specimens were scanned using a Bruker Skyscan 1173 at the Karel F. Liem Bio-Imaging Center at the University of Washington’s Friday Harbor Labs.

### Imaging & comparison of corallivore dentitions

We used SEM to qualitatively examine dentition and soft tissues for a subset of obligate and facultative corallivores (*C. ornatissimus, C. ephippium, C. lunula, H. chrysostomus*, and *F. flavissimus*) for which destructive sampling was allowed. Jaws from formalin-fixed specimens were removed and dehydrated using an ethanol series (24 h 70% ethanol followed by 4 h 100% ethanol), then split into upper and lower dentitions. Jaws were desiccated using a critical point dryer (Samdri 790, Tousimis Research Corp., Rockville, MD, USA) and sputter coated with silver using a Cressington 108 Sputter Coater (Ted Pella, Inc, Redding, CA, USA). Samples were mounted with the anterior surface facing upwards and imaged using a JEOL Neoscope JCM-5000 (JEOL Ltd., Tokyo, Japan) and a Zeiss Evo 10 (Carl Zeiss AG, Oberkochem, Baden-Württemberg, Germany) at 15vK.

### Linear morphometrics

**Fig. 1:**
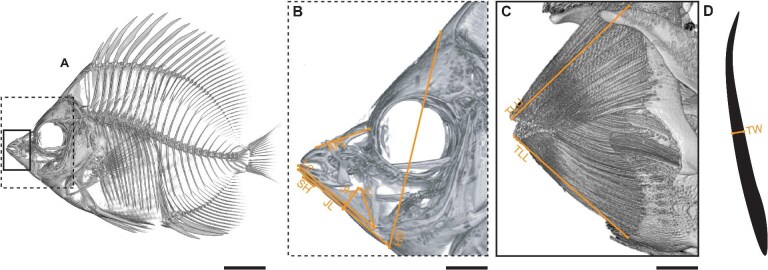
(A) Micro-CT rendering of *Chaetodon melannotus* showing linear morphometric measurements of (B) cranial and (C, D) dentition traits. Schematic depicts cross-sectional tooth-width measurement (D). Trait measurements shown (HD, head depth; JH, jaw height; SH, symphyseal height; TLU, tooth length upper; TLL, tooth length lower; TW, tooth width; APL, ascending process length; Lo, out lever; and Li, in lever). Scale bars correspond to 2 cm, 1 cm, and 3 mm, respectively.

We used 3D Slicer (version 5.10.0) (Fedorov et al. 2012) with the SlicerMorph extension (Rolfe et al. 2021) to take 13 linear measurements (Fig. 1) from μCT renderings to investigate whether functional traits differed between obligate, facultative, and non-corallivorous species. These measurements included head depth (HD), jaw width (JW), lower jaw length (JL), lower jaw height (JH), symphyseal height (SH), ascending process length (APL), jaw opening lever (Lo), jaw closing lever (Li), lower dentition width (DWL), upper dentition width (DWU), lower tooth length (TLL), upper tooth length (TLU), and tooth width (TW). HD was measured as the distance between the first dorsal spine and the posteriormost tip of the dentary. JW was measured between the widest point of the upper and lower jaws. JL was measured as dentary length, and JH measured at the maximum height of the dentary. SH was measured along the suture line of the mandibular symphysis. APL was measured from the posteriormost tip of the ascending process to the margin of tooth attachment. Tooth measurements were taken directly from the surface of the scan without segmentation. Anterior teeth were defined as those immediately above the mandibular symphysis, which were fully visible on the external surface without the need to isolate or remove overlying structures. Where teeth were present in rows, only the outermost row was measured. TL was measured at the anteriormost point on the upper and lower jaws as a linear distance from the base of the tooth, where it attaches to the jaw, to the tip of the tooth cusp; this measure ignores curvature of the tooth but can be applied consistently across species (Fig. 1). TW was measured at the midpoint of the tooth shaft from three of the anteriormost teeth (e.g., at the symphysis) that were rendered most clearly and averaged. We also quantified mechanical advantage (MA) as the ratio Li/Lo. To convert raw measurements into values independent of specimen size, we used log-shape ratios (Claude 2013). Each measurement was first divided by the geometric mean of JL, JW, and HD, then log transformed. Mechanical advantage (MA) was log-transformed without size standardization.

### Statistical analysis of feeding morphology

To identify major axes of morphological variation between species of Chaetodontidae along a coral-dependency gradient (obligate, facultative, and non-corallivore), we used principal components analysis (PCA). We employed two complementary methods to make quantitative comparisons of feeding morphology between groups. First, we conducted phylogenetic ANOVA tests using the residual randomization in a permutation procedure method of (Collyer and Adams 2018) implemented in the R package “*geomorph*” (Baken et al. 2021; Adams et al. 2025) to ask if univariate functional traits differed between trophic guilds (*n* = 9999 permutations for each test). We applied a Bonferroni correction to *P*-values to account for multiple comparisons and used the “*pairwise*” function for post-hoc significance tests. Second, we created *n*-dimensional hypervolumes to compare the volume, degree of overlap, and uniqueness of the multivariate morphospace of obligate and combined facultative and non-corallivorous species using the R package “*hypervolume*” (Blonder et al. 2014, 2018). We first created volumes for each group using Gaussian kernel density estimation; the first 3 PC axes (accounting for 94% of variance) were used as input. We then created a sample of 1000 permuted volumes and generated a null distribution of overlap statistics under the null hypothesis that the two groups occupy the same volume of 3-dimensional morphospace. We estimated the total volume, fraction of space unique to each group, and Jaccard and Sorensen similarity indices, which quantify the degree of overlap between group volumes (Blonder et al. 2018). *P*-values for the statistics were calculated with respect to the generated null distribution.

## Results

### The evolution of corallivory across coral reef fishes

**Fig. 2:**
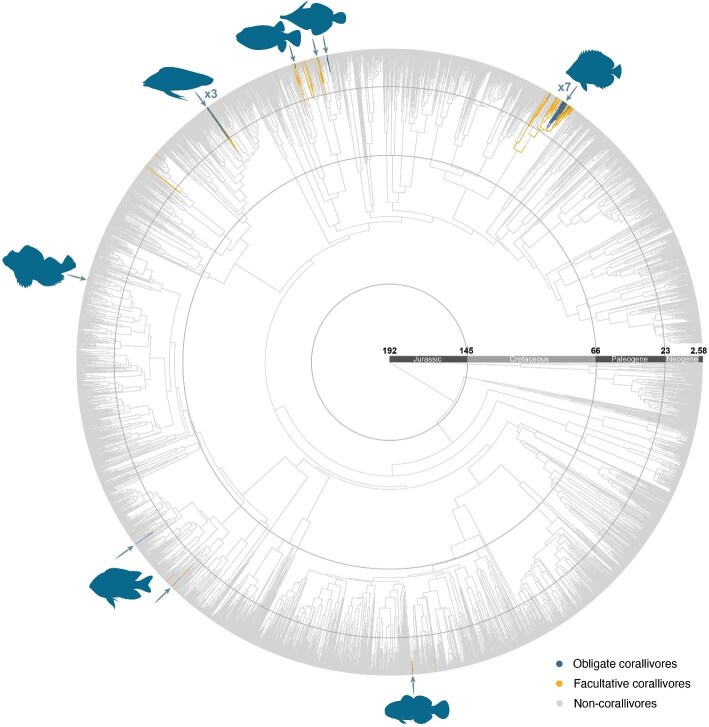
Maximum *a posteriori* (MAP) ancestral state reconstruction of facultative and obligate corallivory across coral reef fishes (*n* = 6175 species). Corallivorous lineages are blue, facultative lineages are gold, and non-corallivores are gray. Silhouettes correspond to major groups in which obligate corallivory has evolved; arrows indicate independent origins of obligate corallivory. Families (clockwise): Chaetodontidae, Gobiidae, Pomacentridae, Blenniidae, Labridae, Tetraodontidae, and Monacanthidae.

**Fig. 3:**
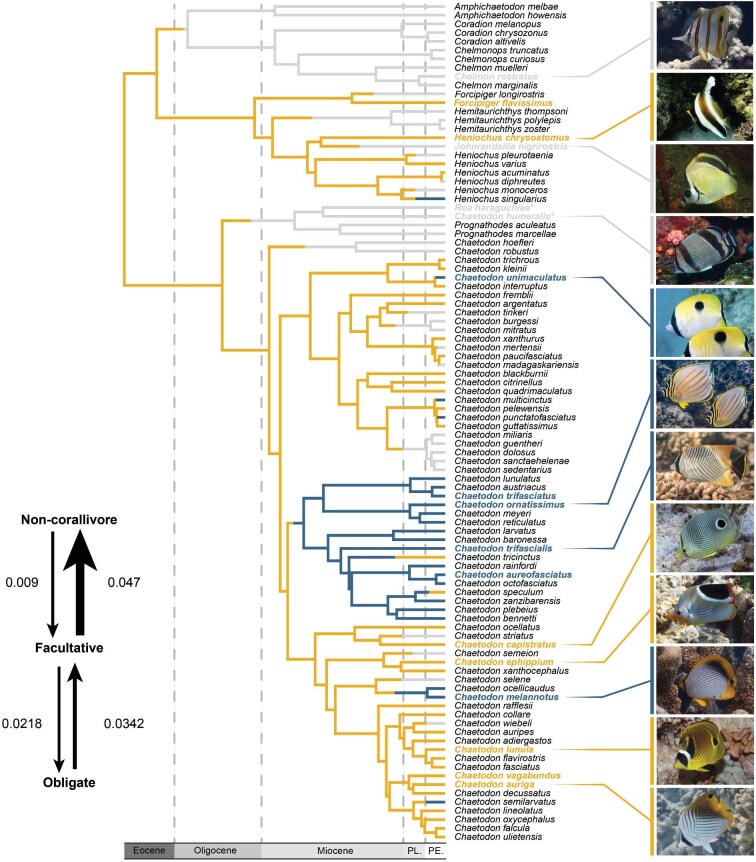
Maximum *a posteriori* (MAP) ancestral state reconstruction of facultative (gold) and obligate (blue) corallivory in Chaetodontidae; non-corallivores are gray. Colored species names correspond to the species included in this study. The arrows and inset indicate the directionality of evolutionary transitions, and the corresponding rate in lineages per million years. PL; Pliocene, PE; Pleistocene. Asterisks (*) mark species substitutions (see Methods). Photo credits: Mike Bowie, CC BY 4.0, https://creativecommons.org/licenses/by/4.0, mirrored. Laszlo Ilyes, CC BY 2.0, https://creativecommons.org/licenses/by/2.0, cropped. Dan Schofield, CC BY 4.0, https://creativecommons.org/licenses/by/4.0, mirrored. Leanard Low, CC BY 2.0, https://creativecommons.org/licenses/by/2.0, Francois Libert, CC BY-SA 2.0, https://creativecommons.org/licenses/by-sa/2.0. Diego Delso, CC BY-SA 4.0, https://creativecommons.org/licenses/by-sa/4.0. Steve Evans, CC BY 2.0, https://creativecommons.org/licenses/by/2.0, mirrored. Brian Gratwicke, CC BY 2.0, https://creativecommons.org/licenses/by/2.0. Isabella Marrable, CC BY 4.0, https://creativecommons.org/licenses/by/4.0.

Feeding on coral mucus, tissue and skeleton is an exceptionally rare ecological niche across coral reef fishes (Fig. 2). The maximum *a posteriori* (MAP) ancestral state reconstruction reveals that obligate corallivory has evolved at least 17 times, both from facultative (*n* = 15 transitions) and non-corallivorous (*n* = 2 transitions) lineages. These direct transitions to obligate corallivory occur in *Exallias brevis* and *Cheiloprion labiatus*. While two reversions from obligate corallivory back to facultative corallivory occur, indicating this trophic niche may not be an evolutionary dead-end (posterior probability [PP] = 0.735), both transitions occur within Chaetodontidae (Figs. 2, 3). No reversions from obligate corallivory occur in other clades of reef fishes. Independent origins of obligate corallivory occur in Gobiidae (*n* = 1), Pomacentridae (*n* = 2), Blenniidae (*n* = 1), Labridae (*n* = 3), Tetraodontidae (*n* = 1), Monacanthidae (*n* = 2), and Chaetodontidae (*n* = 7). Facultative corallivory evolves and is lost more frequently, with 25 transitions into the niche from non-corallivorous lineages and 27 reversions back to a non-corallivore state. The rate at which facultative corallivory evolves (0.00039 transitions/My) is nearly 38× lower than the rate at which it is lost (0.0149 lineages/My). The rate of obligate corallivory directly evolving from a non-corallivorous state (0.00003 transitions/My) is 204× lower than the rate of obligate corallivory evolving from a facultative state (0.0061 transitions/My). Coral reef fishes have spent just 1.4% of their evolutionary history in a state of either facultative or obligate corallivory (Fig. 2). The MAP reconstruction places the first origination of corallivory in any form roughly ∼42 Ma in the ancestor of extant butterflyfishes (Chaetodontidae) (Fig. 2), but early origins also occur in Monacanthidae, Balistidae, and Labridae between 40 and 30 Ma.

Patterns of the evolution of corallivory differ within Chaetodontidae when compared to all reef fishes. Within Chaetodontidae, lineages move through a state of facultative corallivory before obligate corallivory evolves; direct transitions from non-corallivore to obligate corallivore do not occur (PP 0 = 0.821). Likewise, the complete loss of corallivory from an obligate state does not occur and this transition must first pass through a facultative state (PP 0 = 0.75). These reversions from obligate to facultative corallivory are rare but do occur (PP 0 = 0.004) at a relatively high rate (0.0342 transitions/My), in *Chaetodon tricinctus* and *C. speculum* (Fig. 3). The MAP ancestral state reconstruction recovered the ancestor of butterflyfishes as a facultative corallivore (Figs. 2 and 3) with a further seven transitions from facultative to obligate corallivory occurring within the group. Reversions from facultative corallivory back to no corallivory are common (*n* = 18); this rate (0.047 transitions/My) is 5.2× faster than the rate at which facultative corallivory evolves (0.009 transitions/My). Similarly, the rate at which obligate corallivory is lost (0.0342 transitions/My) is 1.6× higher than the rate at which obligate corallivory evolves (0.0218 transitions/My). Obligate corallivores are predominantly found in a single clade of *Chaetodon*, but this feeding mode has also evolved independently within *Heniochus (H. singularius)* (Fig. 3). The ancestor of Chaetodontidae was most likely a facultative corallivore, and 46% of the evolutionary history of this group was spent in a facultative state (Figs. 2 and 3).

### Morphology of obligate, facultative, and non-corallivorous chaetodontids

**Fig. 4:**
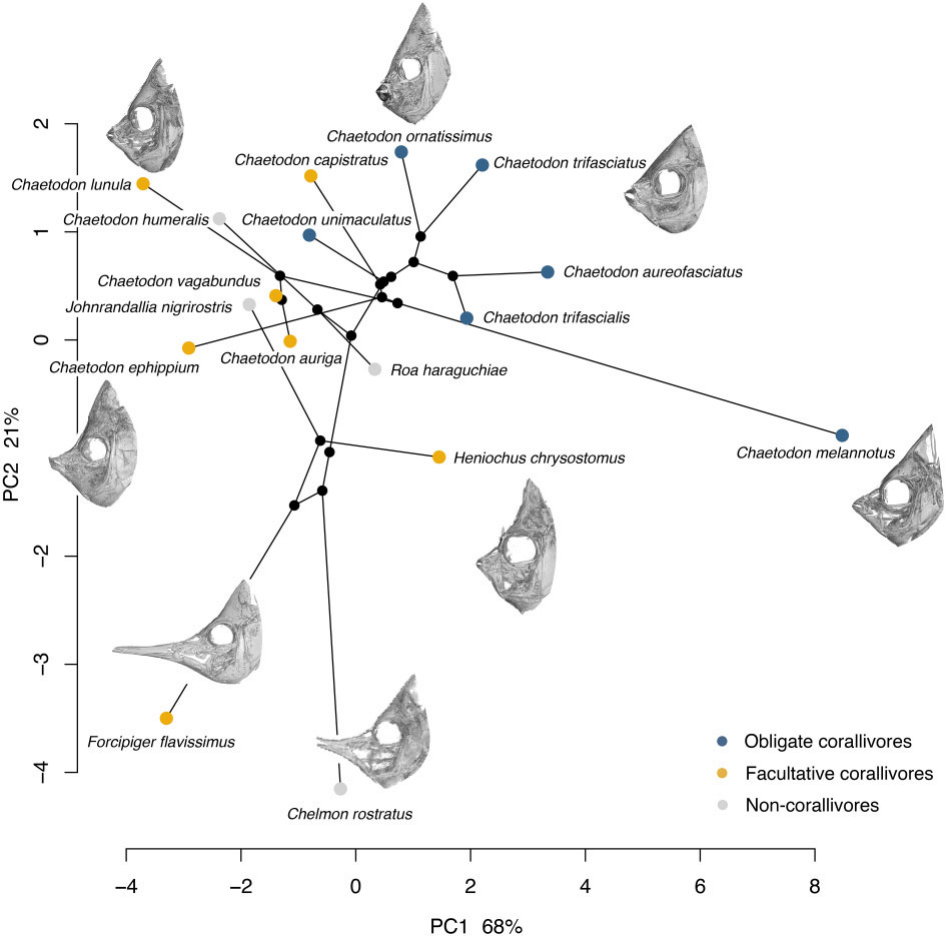
Phylomorphospace of functional feeding traits. Principal component biplot with phylogeny overlaid; obligate corallivores are blue, facultative corallivores are gold, and non-corallivorous species are gray. 3D micro-CT renderings correspond to the species at the extremes of the morphospace.

**Fig. 5:**
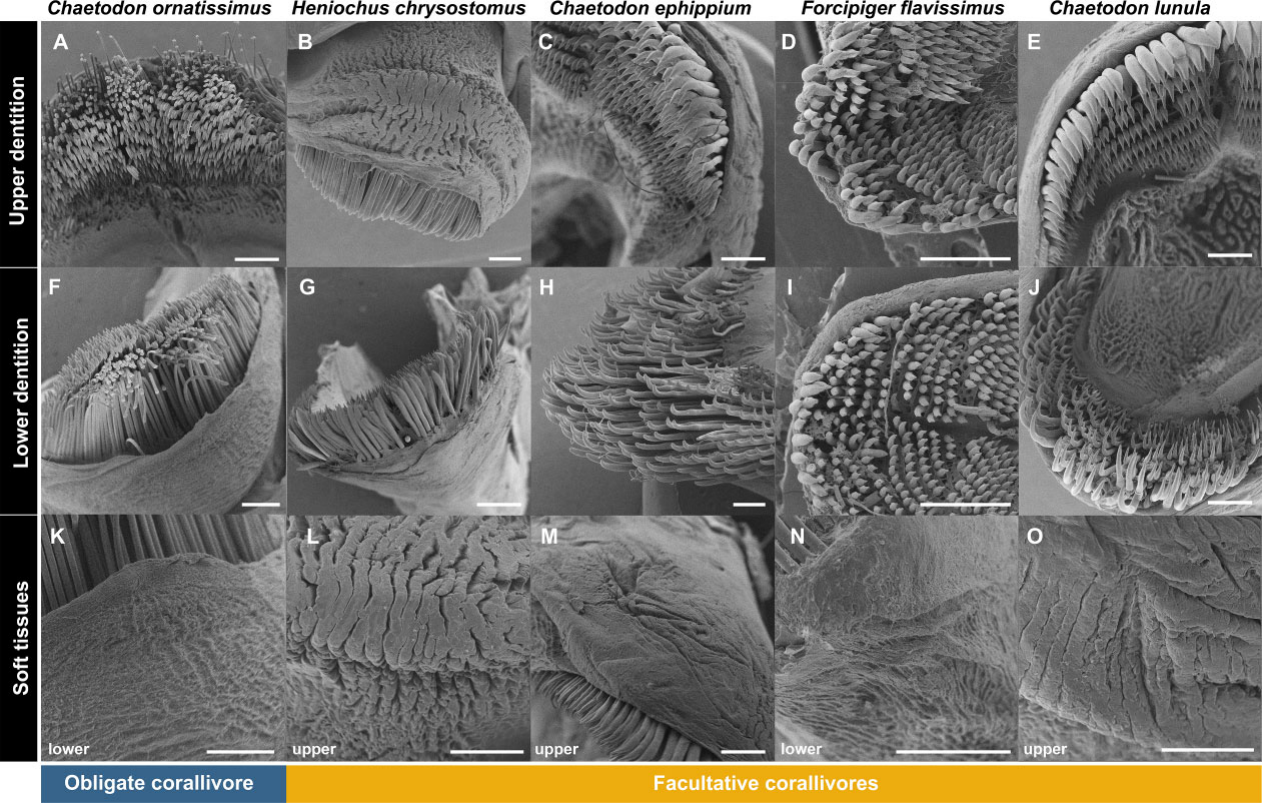
Scanning electron microscopy (SEM) imaging of obligate (blue) and facultative (gold) corallivore dentition and soft tissue traits. High resolution SEM images represent a subset of species considered in broader morphological comparisons. Scale bars correspond to 500 µm.

Principal component analysis shows that obligate corallivores segregate in morphospace from both facultative and non-corallivorous species, while facultative and non-corallivorous species overlap (Fig. 4). PC1, which accounts for ∼68% of the variance in the data, separates some obligate species from facultative and non-corallivores (Fig. 4). Obligate corallivores have shorter heads with wider, taller jaws. The dental bands in obligate corallivores are wider and their teeth are longer than facultative corallivores and species that do not consume coral (Figs. 4 and 5, Fig. S1). PC2 (∼21% variance) accounts for variation in symphyseal height, the length of the ascending process, and mechanical advantage. Although these traits do not show consistent patterns between trophic guilds (Table 1), obligate corallivores do tend to have lower mechanical advantage. Individual comparison of traits with phylogenetic ANOVA indicates that dental traits, rather than cranial traits, differ significantly between trophic guilds, as well as jaw width (Table 1). However, no post-hoc pairwise comparisons were significant due to the small sample size of each trophic guild. The morphospace volumes of obligate and facultative/non-corallivores occupy different total volumes with considerable overlap (Table 2). Jaccard and Sorensen similarity values are 0.16 and 0.27, respectively, indicating a degree of similarity between the two morphospaces. The fraction of unique morphospace is higher, but not significantly so, for facultative and non-corallivorous species (Table 2).

**Table 1:** Phylogenetic ANOVA results comparing dentition and cranial morphometric traits (HD, head depth; LDW, lower dentition width; UDW, upper dentition width; JW, jaw width; JH, jaw height; SH, symphyseal height; TLU, tooth length upper; TLL, tooth length lower; TW, tooth width; APL, ascending process length; Lo, out lever; Li, in lever; and MA, mechanical advantage) between obligate, facultative, and non-corallivorous species. Bonferroni adjusted *P*-values are reported

Trait	*F*-value	*Z*-value	*R*^2	*P*-value	Category
HD	4.371	1.866	0.384	0.364	Cranial
**LDW**	**13.958**	**3.458**	**0.666**	**0.0091**	**Dentition**
**UDW**	**9.312**	**2.87**	**0.571**	**0.0247**	**Dentition**
**JW**	**8.216**	**2.696**	**0.539**	**0.0494**	**Cranial**
JH	4.495	1.878	0.391	0.3523	Cranial
SH	1.984	0.917	0.221	2.3205	Cranial
**TLU**	**10.059**	**2.953**	**0.589**	**0.0208**	**Dentition**
TLL	7.428	2.489	0.515	0.0741	Dentition
TW	2.988	1.416	0.299	1.0296	Dentition
APL	1.134	0.377	0.139	4.6059	Cranial
Lo	3.505	1.585	0.334	0.715	Cranial
Li	4.003	1.747	0.363	0.4927	Cranial
MA	0.526	−0.249	0.069	7.7311	Cranial

**Table 2:** Comparison of hypervolume size and similarity between facultative (F) and non-corallivore (NC) and obligate (O) species

	Volume	Fraction unique	Jaccard	Sorensen
F + NC	266.94	0.795 (0.372)	0.161 (0.204)	0.277 (0.204)
O	127.13	0.57 (0.174)	-	-

*Note*: *P*-values are reported in parentheses.

### Soft tissues and teeth

Corallivores exhibit marked diversity in dental morphology across species and between feeding guilds. Facultative corallivores (*H. chrysostomus, C. ephippium, F. flavissimus*, and *C. lunula*) share broadly similar tooth traits, characterized by thick, conical teeth with prominent anterior-facing cusps. The teeth of *H. chrysostomus*, however, contrast the other three species in that they are wider at the cusp than at the base (Fig. 5B, G). In contrast to facultative species, the obligate corallivore *C. ornatissimus* possesses thin, densely packed, flexible teeth that form an interlocking, brush-like surface (Fig. 5A, F). The arrangement and organization of teeth also differs among species. In *F. flavissimus*, the upper and lower jaws are arranged into sixteen and eighteen discrete pads (Fig. S1C, G), whereas in *H. chrysostomus* the teeth are arranged into tightly ordered rows along the perimeter of both jaws (Fig. 5B, G). In *C. ephippium*, the upper jaw has 10 ordered rows of teeth (Fig. S1B, F), while the lower jaw shows a less discrete arrangement, with teeth extending beyond the upper margin and forming a distinct underbite. *Chaetodon lunula* displays seven upper and 10 lower ordered rows spanning a large surface area and extending toward the buccal cavity (Fig. 5J). Gross examination of soft tissues reveals some differences between species. The lips and surrounding epithelium of *H. chrysostomus* (Fig. 5K) and, to a lesser extent, *C. lunula* (Fig. 5O) exhibit distinct folds, which contrasts the smooth lips of the remaining species (Fig. 5K–O).

## Discussion

In this study, we investigated the evolutionary history of facultative and obligate corallivory and assessed if corallivory represents an evolutionary “dead end”. Second, we examined whether feeding morphology differed between butterflyfish species that vary in their reliance on coral as a food resource. Our results suggest that obligate corallivory is an evolutionarily irreversible niche for most lineages of reef fishes and may require modification of the dentition and soft tissues. By interpreting these results in the context of modern climate change, we highlight the vulnerability of corallivorous fishes at ecological timescales.

### The origin and evolutionary dynamics of corallivory on coral reefs

Both facultative and obligate corallivory have evolved multiple times in coral reef fishes; obligate corallivory most often evolves from a state of facultative corallivory (Fig. 2). For most reef fish clades, we find that obligate corallivory represents an evolutionary “dead end,” given that reversions from obligate to facultative corallivory only occur in two species of *Chaetodon* (Figs. 2, 3). The inability to transition away from specialized trophic niches can lead to negative evolutionary outcomes at the clade level as the extinction risk of lineages is heightened with time (Van Valkenburgh 1991; Balisi and Van Valkenburgh 2020; Tyler and Younger 2022). In contrast to obligate corallivores, reversions from facultative to non-corallivory occur frequently at high rates, and we propose two possible mechanisms to explain this pattern. First, facultative corallivores (<80% coral diet) (Cole et al. 2008) incorporate other invertebrates and algae into their diets. Maintaining this diet variability might facilitate quick transitions away from corallivory when changes to the ecological landscape occur or new competitors emerge. Second, the many independent origins and losses of facultative corallivory in phylogenetically distant and phenotypically diverse groups suggest multiple evolutionary pathways exist when transitioning into and out of a facultative corallivorous niche (Fig. 2). This phylogenetic diversity reflects the range of feeding strategies (e.g., scraping, excavating, picking, and mucus-feeding) used to feed on coral (Bellwood and Choat 1990; Cole et al. 2008; Rotjan and Lewis 2008; Floeter et al. 2018). The interaction of these two factors may allow lineages that maintain a facultative level of corallivory to escape the trap of hyperspecialization and quickly revert to other ecological niches, such as feeding on mobile invertebrates, when changes to the ecological landscape occur. In addition, emergence and dispersal of reef fish families as shallow marine environments expanded and contracted (Cowman and Bellwood 2013; Cowman et al. 2017) may have provided multiple opportunities for corallivory to evolve in the absence of existing corallivorous species.

Our ancestral state reconstruction of corallivory across all extant reef fishes places the first origin of any corallivory ∼42 Ma, in the ancestor of extant butterflyfishes and bannerfishes (Fig. 2). By examining the phylogenetic distribution and evolution of this niche at broad scale, rather than within individual groups, our results challenge the origins of corallivory from previous studies, which suggested tubelip wrasses to be the first corallivores ∼29 Ma (Cowman et al. 2009; Bellwood et al. 2010; Huertas and Bellwood 2018). Tubelip wrasses were likely the first obligate corallivores, but our results suggest facultative corallivory may have evolved earlier (Fig. 2). However, it is also possible that older lineages may have engaged in obligate or facultative corallivory before going extinct. Our results indicate that facultative corallivory is necessary precursor to the evolution of obligate corallivory for most lineages (Fig. 2). Early origins of facultative corallivory also occur in Tetraodontiformes, which have robust jaws specialized for biting, hypertrophied adductor mandibulae muscles, and large teeth (Turingan and Wainwright 1993; Burns et al. 2024). Tetraodontids and balistids ingest portions of coral skeletal material following bites on the coral surface and are also known to directly bite off the tips of branching corals (Hiatt and Strasburg 1960; Sano et al. 1984). Most extant species are facultative corallivores (Cole et al. 2008), suggesting their existing durophagous feeding morphology may have been co-opted for coral feeding while enabling the incorporation of other primary durophagous prey items, such as molluscs and crabs. Durophagous morphology, which also induced ecological diversification in wrasses (Labridae), may therefore be an exaptation for facultative corallivory because hypertrophied musculature and robust skeletal elements likely provide an advantage for scraping and excavating feeding strategies (Gould and Vrba 1982; Burress and Wainwright 2019). These patterns highlight the multiple evolutionary pathways to corallivory, as families that contain corallivorous species differ tremendously in skull and body morphology.

Within butterflyfishes, our results indicate that the ancestor of the group was a facultative corallivore (Figs. 2 and 3). We find that obligate corallivory is a much younger trophic niche, first appearing within *Chaetodon* ∼15 Ma, which agrees with previous studies (Fig. 3) (Bellwood et al. 2010). Transitions into new trophic niches, particularly those associated with the benthos, have had large impacts on the pace of reef fish diversification (Siqueira et al. 2020; Corn et al. 2022; Peoples et al. 2026). As butterflyfishes emerged in a highly-diverse system with many prominent reef fish families already present (Bellwood 1996; Ghezelayagh et al. 2022), the early incorporation of coral into their diets may have contributed to their diversification in competitive reef environments. Their dental morphology of long, bristle-like teeth (Fig. 5) (Motta 1988, 1989) may have been an advantage for coral feeding, although it is unknown if this trait was present in the earliest lineages as representation of early Chaetodontids in the fossil record is limited (Carnevale 2006).

### Corallivore morphology

Previously, it was suggested that corallivory does not increase cranial diversity and rather, specialization of the teeth, lips, and digestive physiology may underlie these trophic transitions (Konow et al. 2017). Our results largely agree with this; we recovered morphological differences between facultative and obligate corallivorous butterflyfishes primarily related to the dentition (Fig. 4; Table 1). The morphospace occupied by facultative and non-corallivorous species was larger than the area occupied by obligate corallivores (Fig. 4, Table 2), a pattern driven by the range of skull morphologies in species that consume little coral (e.g., *Chelmon, Forcipiger*) (Fig. 4). We found no group-differences across all cranial traits measured except jaw width (Table 1). Dental traits are associated with many trophic specializations in fishes, including lower trophic levels (Kolmann et al. 2018; Heiple et al. 2023; Huie et al. 2025; Peoples and Wainwright 2025; Peoples et al. 2025; Williams and Price 2025). The dense pad of long, flexible teeth in obligate corallivores (e.g., *C. ornatissimus*) may provide an advantage when removing mucus and coral tissue, whereas the stouter, curved teeth in facultative corallivores may be beneficial when capturing small elusive prey (Fig. 5) (Motta 1988, 1989). While external morphology relates to resource acquisition, internal morphology and digestive physiology likely plays an equally important role in processing and assimilation as cnidarians are considered a nutrient-poor resource (Berumen et al. 2011). Corallivores have longer and narrower guts than non-corallivores, possibly to facilitate the processing of complex polysaccharides in mucus or manage defensive secondary metabolites in tissue (Alino et al. 1992; Gochfeld 2004; Berumen et al. 2011; Ghilardi et al. 2021). Gut microbiome composition of corallivores also differs from other trophic groups (Degregori et al. 2024) and can change to reflect coral consumption (Clever et al. 2022). Other species that specialise on difficult resources (e.g., collagen-rich fish scales) also have both changes in feeding morphology and digestive physiology (Takahashi et al. 2007; Baldo et al. 2015; Heras and Martin 2022). Taken together, transitions toward difficult, nutrient-poor resources likely require significant coordinated change in both capture and processing morphology as well as digestive physiology.

### The paradoxical evolution of corallivory

Stony corals (Scleractinia) are foundational elements of tropical reef ecosystems and form the primary structural components of coral reefs. While many species of fish recruit to the interwoven branches of coral colonies and depend on the three-dimensional structural complexity for refuge throughout their life histories, far fewer have evolved to feed upon it (Cole et al. 2008; Bellwood et al. 2010) (Fig. 2). The paradox of its abundance in contrast to the rarity of corallivores is intriguing, given that we find multiple independent transitions into corallivorous niches in phylogenetically distant clades (Fig. 2). The asymmetry between coral dominated systems and corallivorous fish diversity is likely, at least in part, due to the inherent challenge of utilizing coral as a trophic resource (Huertas and Bellwood 2017). Coral is made up of a sharp, calcium carbonate skeleton, which houses a thin, living tissue layer that is laden with potent nematocysts. The low nutritional density of coral tissue (Berumen et al. 2011) requires corallivorous fishes to forage actively throughout the day at consistent rates to meet metabolic demands (Gregson et al. 2008), a pattern that contrasts with piscivores, for example, that can feed episodically on nutrient-dense prey. Corallivorous fishes consequently operate with narrow energetic margins, a constraint that has both driven specialized niche partitioning and rendered them vulnerable to resource disturbance (Graham et al. 2011). However, the density of corals on reefs seemingly provides enough of an incentive for a small number of fish lineages to use these cnidarians as resources, as is the case with other low-energy-density foods, such as plants or detritus and their consumers.

### Modern extinction risk for corallivores

In addition to the evolutionary “dead end” of obligate corallivory for most reef fish lineages, corallivores face acute extinction risks on modern reefs. Coral reefs are among the most vulnerable ecosystems, with climate-driven disturbances that alter resource availability occurring with increased frequency (Hoegh-Guldberg et al. 2007; Urban 2015). As thermal anomalies and bleaching events intensify, specialized corallivorous fishes may be unable to adapt quickly enough to avoid population declines and local extinctions (Graham et al. 2011). Obligate corallivores decline following the loss of live coral cover due to their dietary inflexibility (Pratchett 2007), whereas facultative species decline following the loss of three-dimensional habitat structure and resulting increase in exposure to predation (Pratchett et al. 2006; Graham et al. 2007; Clinchy et al. 2013). Climate-driven disturbance can also lead to severe impacts on an organism’s foraging behavior (Candolin and Rahman 2023). Corallivorous fishes weaken dietary preferences in response to resource scarcity, leading to consumption of unpreferred food resources (Berumen et al. 2005; Keith et al. 2018; MacDonald et al. 2021; Semmler et al. 2022). The limited divergence in functional traits between obligate and facultative corallivores (Fig. 4) may contribute to this by not constraining the acquisition of alternate food resources. This flexibility has the capacity to bolster corallivores in response to disturbances at some scales, but may not be enough to prevent population declines following compounding coral bleaching events and widespread reef structural collapse (Graham et al. 2009; Semmler et al. 2025). Combined with physiological constraints, such as a microbiome and digestive morphology that can be destabilized by thermal stress (Berumen et al. 2005; Clever et al. 2022), obligate corallivores face a convergence of stressors accumulating faster than ecological or evolutionary responses can track.

## Conclusion

Our finding that obligate corallivory is an evolutionary “dead end” for most fishes suggests there may be evolutionary consequences following ecological specialization on coral reefs, whereas maintaining more generalized diets (e.g., facultative corallivory) may allow for greater flexibility at ecological and evolutionary scales. This maintenance of dietary versatility, and the multiple origins of moderate corallivory in disparate groups, appear to have allowed facultative corallivores to escape consequences of hyperspecialization while providing ecological novelty in competitive reef environments. Adaptation to corallivorous diets in butterflyfishes is defined by modification to the dentition, digestive morphology, and potentially other more subtle changes to soft tissues rather than by dramatic cranial reorganization. Future investigation of additional corallivorous lineages is warranted to identify shared modifications to the feeding system that enable species to feed on coral. While the dynamic evolutionary history of corallivory reflects transitions on a past ecological landscape, the present and accelerating threat of climate change is increasing the modern extinction risk of species that occupy this rare and specialized ecological niche on coral reefs.

## Supplementary Material

obag032_Supplemental_File

## Data Availability

Scans available on MorphoSource are listed in Table.
