# Supplementary material for: The Evolutionary History and Morphological Divergence of Corallivorous Fishes on Coral Reefs
Source: Integr Org Biol. 2026 Aug 8;8(1):obag032. doi: 10.1093/iob/obag032 (PMC13458402; doi:10.1093/iob/obag032)
Supplement: Supplementary file 1 [file obag032_supplemental_file.docx]

**Supplementary Figures and Tables**

**Figure S1 |** Scanning electron microscopy (SEM) imaging of obligate (blue) and facultative (gold) corallivore tooth arrangement. Dotted lines delineate representative tooth rows and pads (*F. flavissimus*). Scale bars correspond to 500 µm.

**Table S1 |** List of specimens and accession numbers.

| **Species** | **Guild** | **Collection** | **Specimen ID** | **MorphoSource ID** | **Scanning electron microscopy** | **Micro-CT** |
| --- | --- | --- | --- | --- | --- | --- |
| *Chaetodon lunula* | Facultative | Private | None |  | Teeth and soft tissue | Whole body, head and jaws |
| *Chaetodon ephippium* | Facultative | Private | None |  | Teeth and soft tissue | Whole body, head and jaws |
| *Chaetodon ornatissimus* | Obligate | Private | None |  | Teeth and soft tissue | Whole body, head and jaws |
| *Heniochus chrysostomus* | Facultative | Private | None |  | Teeth and soft tissue | Whole body, head and jaws |
| *Forcipiger flavissimus* | Facultative | Private | None |  | Teeth and soft tissue | Whole body, head and jaws |
| *Chaetodon auriga* | Facultative | LACM | 35977.011 | 000653847 | None | Head and jaws |
| *Chaetodon trifasciatus* | Obligate | LACM | 3783.000. | 000654682 | None | Head and jaws |
| *Chaetodon aureofasciatus* | Obligate | LACM | 3783.000. | 000654360 | None | Head and jaws |
| *Chaetodon melannotus* | Obligate | LACM | 9633.003 | 000654321 | None | Head and jaws |
| *Chaetodon trifascialis* | Obligate | LACM | 3976.002 | 000654712 | None | Head and jaws |
| *Chaetodon humeralis* | Non-corallivore | FLMNH | 792152 | 000792155 | None | Head and jaws |
| *Roa haraguchiae* | Non-corallivore | CAS | 792141 | 000792148 | None | Head and jaws |
| *Johnrandallia nigrirostris* | Non-corallivore | FLMNH | 792131 | 000792134 | None | Head and jaws |
| *Chaetodon vagabundus* | Facultative | LACM | 42481.011 |  | None | Head and jaws |
| *Chaetodon unimaculatus* | Obligate | LACM | 38226.005 |  | None | Head and jaws |
| *Chelmon rostratus* | Non-corallivore | Yale Peabody Museum | 425822 | 000426071 | None | Head and jaws |
| *Chaetodon capistratus* | Facultative | Yale Peabody Museum | 425867 | 000426493 | None | Head and jaws |
